# Magnetocaloric Effect and Critical Behavior across the Second-Order Ferromagnetic–Paramagnetic Phase Transition of a NdSmNiMnO_6_ Double Perovskite

**DOI:** 10.1021/acsomega.5c00940

**Published:** 2025-04-30

**Authors:** John M. Attah-Baah, Romualdo S. Silva, Cledson Santos, Maria H. Carvalho, Nilson S. Ferreira

**Affiliations:** † Departamento de Física, Universidade Federal de Sergipe, 49100-000 São Cristóvão, SE, Brazil; ‡ Instituto de Ciencia de Materiales de Madrid (ICMM), CSIC, E-28049 Madrid, Spain; § Departamento de Física, Universidade Federal do Espírito Santo, 29075-910 Vitória, ES, Brazil; ∥ Instituto de Física Gleb Wataghin, Universidade Estadual de Campinas (UNICAMP), 13083-859 Campinas, SP, Brazil

## Abstract

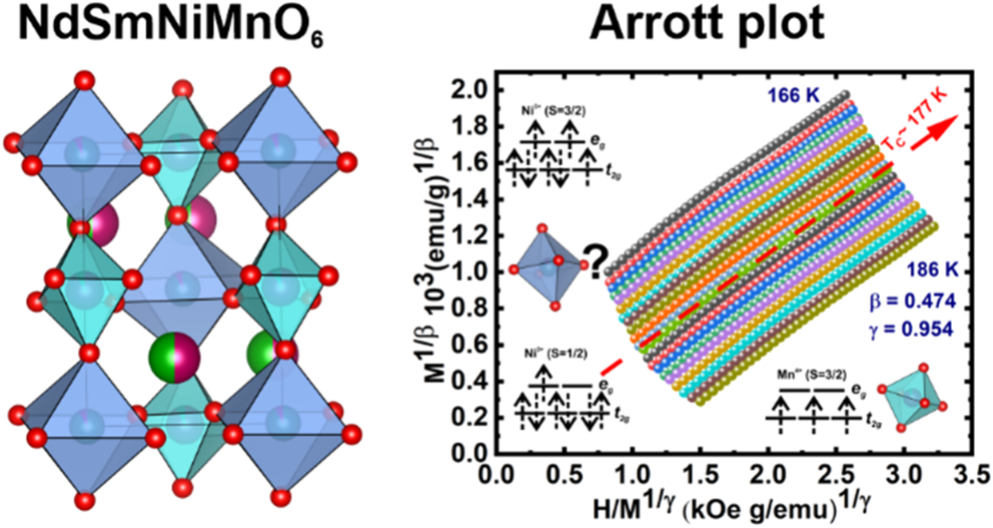

A key challenge remains to improve or discover magnetic solids with optimal magnetocaloric effect (MCE) performance, providing a promising and environmentally friendly cooling technology. Herein, we report the crystal structure, magnetocaloric effect, and critical behavior of double perovskite NdSmNiMnO_6_ synthesized by the modified sol–gel process. X-ray diffraction structural investigation reveals that NdSmNiMnO_6_ crystallizes in the monoclinic *P2*_1_/*n* (14) space group. The magnetocaloric analysis unveils a maximum magnetic entropy change of −Δ*S*_M_^max^ = 2.38 J kg^–1^ K^–1^ (at 0–7 T) near the ferromagnetic to paramagnetic second-order phase transition at 180 K. Furthermore, the estimated relative cooling power value increases from ∼25 to ∼182 kg^–1^ K^–1^ when the applied field changes up to 0–7 T, suggesting a promisor magnetic refrigerant material. The critical behavior investigated by techniques such as the modified Arrott plot, the Kouvel–Fisher method, and the critical isotherm analysis reliably yields critical exponents β = 0.469, γ = 0.978, and δ = 3.09, in agreement with the scaling hypothesis. Lastly, the renormalization group theory analysis revealed a magnetic interaction distance decaying as *J*(*r*) ∝ *r*^–4.63^, which is between the three-dimensional (3D) Heisenberg and the mean-field models, suggesting that the critical behavior of NdSmNiMnO_6_ can be attributed to the competition between long- and short-range magnetic interactions.

## 1 Introduction

The rare-earth (*RE*)-based double-perovskite oxides with general formula *RE*_2_BB′O_6_, where *B* and *B*′ are transition metal ions, are derived from the canonical perovskite oxide ABO_3_.^1−3^ The aristo-type or idealized perovskite structure has a cubic symmetry with a *Pm*3̅*m* representative space group, where the octahedral cation sublattice adopts a NaCl-like topology, which is the most prevalent form of B-cation ordering in perovskites. This 1:1 rock-salt ordering of B-site cations doubles the unit cell of the undistorted ABO_3_ perovskite, transitioning its space group symmetry from *Pm*3̅*m* to *Fm3̅m*.^2,4^ They adopt either an orthorhombic or a monoclinic crystal structure. Both maintain patterns of octahedral tilts to satisfy constraints of the tolerance factor. The orthorhombic structure has chemical and charge disorder at the B sites, while in the monoclinic case, B site cations are ordered by both chemical species and charge (e.g., B^2+^ and B^′4+^).^5^ Its Glazer tilt system is typically described as *a*^–^*a*^–^*b*^+^, where the adjacent octahedra show antiphase tilting along the *a*- and *b*-axes (*a*^–^) and in-phase tilting along the *c*-axis (b^+^).^4,6−8^ This tilt configuration reduces symmetry, leading to monoclinic distortions caused by the antiphase tilts along the *a*- and *b*-axes. In contrast, the in-phase tilts along the *c*-axis maintain partial coherence, contributing to the monoclinic symmetry observed in this case. These combined distortions affect bond angles and lengths, influencing the material’s magnetic, electronic, and structural properties.^9^ Most ordered double perovskites exhibit ferromagnetism (FM), which arises from superexchange (SE) interactions between the ordered *B*^*2+*^ and *B*^′4+^ ions; however, the antisite disorder (ASD) of the B-site cations can have a profound impact on the magnetic state.^5^ In the case of nominally cation-ordered La_2_NiMnO_6_, antiferromagnetic (AFM) Ni^2+/3+^–O–Ni^2+/3+^ and Mn^4+/3+^–O–Mn^4+/3+^ SE interactions induced by ASD coexist and compete with FM coupling attributed to Ni^2+^–O^2–^–Mn^4+^ interactions.^10−12^

Moreover, magnetic refrigeration (MR) harnesses magnetocaloric effects (MCEs) in magnetic materials, presenting a promising, eco-friendly cooling technology. A key challenge remains of enhancing or discovering magnetic solids with optimal MCE performances to bridge the gap between research discovery and practical implementation. Thus, investigation of magnetic properties and MCE in *RE*_2_BB′O_6_ DP oxides with ordered B-site atom structure, for example, Gd_2_ZnMnO_6_, exhibits outstanding MCE performance with magnetic entropy change *–*Δ*S*_M_^max^ ∼ 25.2 J/kg K around 6.4 K under Δ*H* of 0–7 T.^13^ Also, Zhang et al.^14^ observed a remarkable reversible cryogenic magnetocaloric effect (MCE), coupled with outstanding MCE performances, in Gd_2_TiMgO_6_, where the −Δ*S*_M_^max^ and refrigerant capacity (RC) were calculated to be 46.21 J/kg K and 300.27 J/kg, respectively, at an approximately 3.3 K under a magnetic field range of 0–7 T. These values notably surpass those of many recently reported renowned cryogenic MC materials and even outperform the commercialized magnetic refrigerant gadolinium gallium garnet (GGG). Although magnetic materials with first-order phase transitions typically exhibit a large magnetocaloric effect (Δ*S*_M_), this effect is confined to a narrow temperature range near the phase transition and is often accompanied by hysteresis, resulting in a low cooling power. To address these limitations, materials with second-order phase transitions are preferred as magnetocaloric candidates. These materials lack thermal hysteresis and exhibit smaller peak entropy changes (Δ*S*_M_^max^), but their significant entropy variation over a broader temperature range provides higher net cooling power.^15,16^

While bulk and thin film configurations of La_2_NiMnO_6_ have been extensively studied, exploration into other rare-earth double perovskites (*RE*_2_NiMnO_6_) remains relatively limited, particularly AA′BB′O_6_. To the best of our knowledge, no work has been performed on NdSmNiMnO_6_. The substitution of Sm^3+^ for Nd^3+^ in this compound is motivated by fundamental considerations such as crystal stability, modifications to the electronic band structure, and enhanced magnetoelectric coupling.^17,18^ The A-site cation (Nd^3+^, Sm^3+^) significantly influences the B-site (Ni/Mn) sublattice, directly impacting magnetic and transport properties.^19^ Structural stability is quantified by the Goldschmidt tolerance factor, *t*_G_ (eq 1), where *r*_A_ (Nd, Sm), *r*_B_ (Ni, Mn), and *r*_O_ (O) are the ionic radii of A-site cations, B-site cations, and oxygen, respectively.^20^ The slight ionic radius difference between Nd^3+^ (1.109 Å) and Sm^3+^ (1.079 Å) alters, affecting Ni–O–Mn bond angles, bond lengths, orbital hybridization, and superexchange interactions.^21^ Sm doping induces chemical pressure, enhancing octahedral tilting and lattice distortions, which modify the electronic bandwidth and charge transport. Also, magnetic interactions arise from competing FM and AFM SE, governed by the Goodenough–Kanamori–Anderson (GKA) rules.^17,22,23^ The primary exchange pathways are FM 180° (Ni^2+^–O–Mn^4+^) and AFM (Ni^3+^–O–Mn^3+^).^24^ Furthermore, Sm substitution induces A-site disorder, leading to local charge fluctuations and modified Ni/Mn valence states, which impact double-exchange mechanisms.^25^ This disorder can introduce spin frustration and phase competition, potentially resulting in glassy magnetic states, as observed in related perovskite.^26^ Additionally, the magnetocaloric effect (MCE) in double perovskites depends on magnetic entropy changes (Δ*S*_M_), optimized by tuning the Curie temperature (*T*_C_) via Sm-induced lattice distortions, enhancing entropy-driven magnetic phase transitions through Ni^2+^–Mn^4+^ interactions, and introducing anisotropic exchange interactions for improved refrigerant capacity.^27−29^ Finally, the mixed valence states (Ni^2+/3+^ and Mn^3+/4+^) enhance the spin polarization, making NdSmNiMnO_6_ a promising candidate. Hereafter, we present a robust investigation of the crystal structure, magnetocaloric effects (MCEs), and critical behavior of the ordered double perovskite NdSmNiMnO_6_.

## 2 Experimental Procedures

### 2.1 Synthesis

The polycrystalline double perovskite NdSmNiMnO_6_ was prepared by a modified sol–gel method, using glycine to accelerate intrinsic combustion. In a sequential synthesis process, 2 g of glycine was slowly added to a 2 M starting solution containing Nd(NO_3_)_3_·6H_2_O, Sm(NO_3_)_3_·6H_2_O, Ni(NO_3_)_2_·6H_2_O, and (CH_3_COO)_2_Mn·4H_2_O, all with high-purity (Aldrich, 99,97%), in a ratio of 1:1:1:1 (Nd/Sm/Ni/Mn). The prepared sample solutions were kept at 200 °C for 24 h on a hot plate for evaporation and xerogel formation. Finally, all of the swollen xerogels were ground and calcined at 1000 °C for 12 h to remove all organic solvent substrates. Furthermore, these samples were subjected to successive pulverization and pressed at a pressure of 80 MPa. Again, the final calcination was carried out in an oven with an ambient atmosphere at 1200 °C/24 h to help form the phase of the systems or obtain the pure double perovskite complexes.

### 2.2 Characterization

The structural characterization of NdSmNiMnO_6_ DP was performed by X-ray diffraction (XRD) using a Rigaku diffractometer, operating with a Cu tube (*Kα*_1_ = 1.540598 Å). In this case, the following conditions were considered: scan of 5–90° 2θ, 40 kV, 40 mA, step 0.02° in 2θ and time/step of 20 s, fixed slit 1/4°, and antiscattering 1/2°, mask 10 mm. The Rietveld refinement^30^ of the whole patterns was carried out using the Fullprof software,^31^ with the procedure outline in ref (32). The magnetic investigations were conducted utilizing a superconducting quantum interference device (SQUID) magnetometer (MPMS-3, Quantum Design) within the temperature range of 2–300 K, with a maximum applied magnetic field (H) of ±70 kOe. To ensure minimal bias from any trapped flux within the superconducting magnet, appropriate demagnetization protocols were employed, wherein the magnetic field was oscillated during the field decrease process, resulting in negligible trapped flux. The chamber temperature was routinely brought to 300 K, surpassing the critical temperature of the superconducting coils before commencing a new set of experiments in the MPMS-3. Additionally, the coils were cooled by helium infusion before the temperature-dependent magnetization (M(T)), and isothermal magnetic field-dependent magnetization (M(H)) measurements were performed.

## 3 Results and Discussion

### 3.1 Structure Characterization

**Figure 1:**
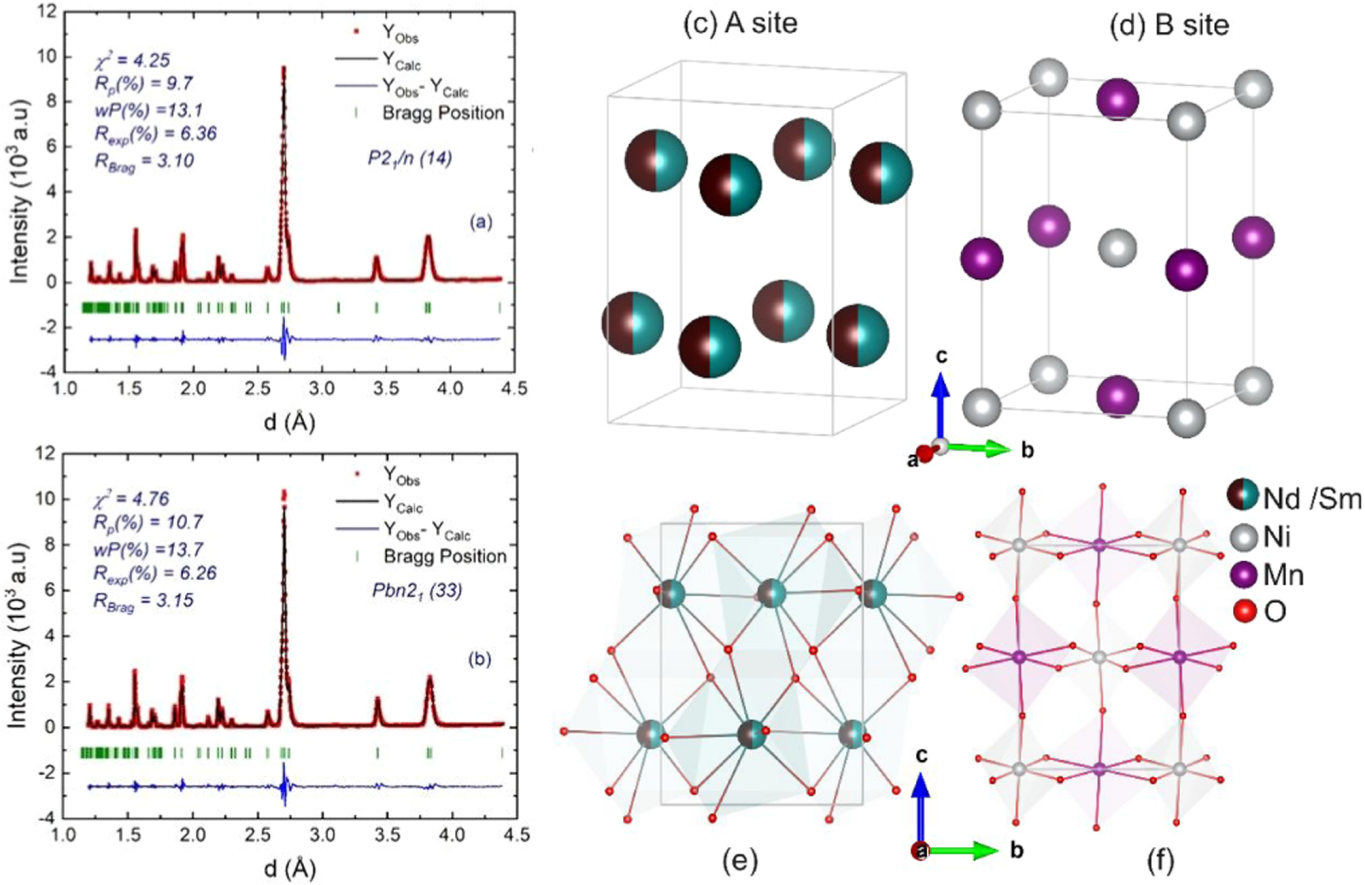
(a, b) Room-temperature XRD and their Rietveld refinements for the NdSmNiMnO_6_ double perovskite. The red square symbols and black line denote the observed and calculated intensities, respectively. Short verticals (green) indicate the position of the possible Bragg reflections of the *P2*_1_/*n* (14) monoclinic and *Pbn2*_1_ (33) orthorhombic structures, respectively. The difference between the observed and calculated profiles (blue) is plotted at the bottom. (c–f) Crystal structure of NdSmNiMnO_6_. Single unit cell in light-gray lines, and the cation–oxygen coordination is shaded in panes.

The XRD pattern for the NdSmNiMnO_6_ powder sample, as obtained experimentally and refined by using the FULLPROF software, is presented in Figure 1a,b. In the refinement process, atomic positions and Debye–Waller factors were iteratively refined over multiple cycles using a pseudo-Voigt function modified by Thompson–Cox–Hastings,^33^ which accounted for axial divergence asymmetry. The refinement was conducted using the conventional orthorhombic symmetry *Pbnm* (62) (not shown), *Pbn2*_1_ (33), and the monoclinic space group, where *Pbnm*/*Pbn2*_1_ possess random B sites and *P2*_1_/*n* has ordered B sites. A more accurate fitting was achieved with the *P2*_1_/*n* (14) space group, indicating that the NdSmNiMnO_6_ crystallizes with the monoclinic space group symmetry, presenting lattice parameters of *a* = 5.3779 (2) Å, *b* = 5.4849(2) Å, *c* = 7.63667(2) Å, β = 89.8412(3)°, and *V* = 225.260(1) Å^3^. In this case, Nd/Sm shared the same crystallographic A-site and coordinates with eight oxygens, forming polyhedrons (Figure 1c,d), whereas Ni/Mn cations are distinctly distributed at the BB′ sites, coordinates with six oxygens, giving rise to an octahedral shape (Figure 1e,f). Moreover, the absence of any discernible impurity peaks within the resolution limits of the instrument for the NdSmNiMnO_6_ sample shows the presence of a highly pure, single-phase material. Moreover, the goodness of fit, as expressed by *R_p_* (%), *R_wP_* (%), *R_exp_* (%), and χ^2^, is determined, and the obtained Rietveld parameters with the corresponding Wykoff sites are summarized in Table 1, confirming a good agreement between the refined and the experimental XRD patterns.

**Table 1:** Structural Parameters of the NdSmNiMnO_6_ Sample at Room Temperature Obtained through the Rietveld Refinement from the XRD Data^a^

NdSmNiMnO_6_ (*P*2_1_/*n*, #14)					
Atom	Frac. coord.	*U*_iso_ (×10^–3^ Å^2^)	BVS|e|	Occ.	Wyck. Pos.
Nd/Sm	*x* = 0.0092(6)	0.12(1)	+3.40/1.73	Nd/Sm	4e
	*y* = 0.5491(2)				
	*z* = 0.75047(8)				
Ni	*x*, *y*, *z* = 0	0.31(2)	+2.88	76%Ni, 24%Mn	2a
Mn	*x* = 0.5	0.31(2)	+3.98	76%Mn, 24%Ni	2b
O1	*x* = 0.0907(3)	1.26(2)	-	O	4e
	*y* = 0.0202(2)				
	*z* = 0.2384(6)				
O2	*x* = 0.2029(5)	0.42(1)	-	O	4e
	*y* = 0.2552(5)				
	*z* = −0.0477(3)				
O3	*x* = 0.2186(5)	1.42(2)	-	O	4e
	*y* = 0.3341(5)				
	*z* = 0.55093(3)				
Bond Length (Å)				Bond Angle (deg)	
Ni–O1	1.89(5)			**<O1–Ni–O1>**	180.00(0)
Ni–O2	1.81(3)			**<O1–Ni–O2>**	89.9(3),90.1(3)
Ni–O3	1.81(3)			**<O1–Ni–O3>**	89.0(10),91.0(10)
Mn–O1	2.06(5)			**<O1–Mn–O1>**	180.00(0)
Mn–O2	2.12(3)			**<O1–Mn–O2>**	88.6(8),91.4(8)
Mn–O3	2.21(3)			**<O1–Mn–O3>**	89.9(8),90.1(8)
				**<Ni–O1–Mn>**	150.5(9)
				**<Ni–O2–Mn>**	155.3(5)
				**<Ni–O3–Mn>**	145.6(5)

a Bond valence sum (BVS) was determined using the parameters, *R*_0_ (Nd^3+^) = 2.49, *R*_0_ (Sm^2+^) = 2.24, *R*_0_ (Ni^3+^) = 2.04, *R*_0_ (Mn^4+^) = 1.71, and *B* = 0.37: BV = exp[(*R*_0_ – *R*)/*B*]. NB: For the mixed occupancy sites, we have the BVS of the majority cation.

Additionally, the stability and deformation of the perovskite crystal structure can be determined through the calculation of the Goldschmidt tolerance factor, *t*_*G*_, utilizing the provided modified formula:
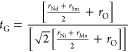
1where *r*_Nd_, *r*_Sm_, *r*_Ni_, *r*_Mn_, and *r*_O_ are the ionic radii of Nd, Sm, Ni, Mn, and O from the Shannon tables, respectively.^25,34^ The calculated ***t***_**G**_ values for NdSmNiMnO_6_ at room temperature, considering Ni^2+/3+^/Mn^3+/4+^ (VI coordination) in the low-spin (LS) and high-spin (HS) states, are 0.834 and 0.862, respectively, which are within the global values, empirically expected to be monoclinic or orthorhombic space group.^25,35,36^ The local crystal structural analysis reveals nonidentical bond lengths and bond angles, specifically, Ni–O1 ≠ Ni–O2 = Ni–O3 ≠ Mn–O1 ≠ Mn–O2 ≠ Mn–O3 and <Ni–O1–Mn> ≠ <Ni–O2–Mn> ≠ <Ni–O3–Mn> ≠ 180°, as outlined in Table 1. This observation hints at the presence of a distorted crystalline structure within the ordered NdSmNiMnO_6_ system. The structural refinement yields an average bond length of 1.84(4) and 2.13(4) Å for *<*Ni–O> and <Mn*–*O>, and an average bond angle of <Ni/Mn–O–Ni/Mn> = 150.50(6)°, respectively. These values align with those obtained for other perovskite systems.^37−40^ Employing the refined lattice parameters obtained, the octahedral rotation angles (γ, φ, Φ) were estimated using the following formulas: γ = cos^–1^(*a*/*b*) ∼ 11.34°, φ = cos^–1^(√2*a*/*c*) ∼ 5.18°, and Φ = cos^–1^(√2*a*^2^/*bc*) = cos^–1^(cosγcosφ) ∼ 12.45° and *Θ = [180*–*<Ni–O–Mn>]/2* ∼ 14.77°. These angles quantitatively describe the extent of octahedral rotation in the perovskite structure, providing valuable insights into its deviation from the ideal cubic configuration. Naturally, disorders such as crystal structure distortion in perovskites are signatures of the emerging magnetic competition and frustration effects in these materials

### 3.2 Magnetocaloric Effect

**Figure 2:**
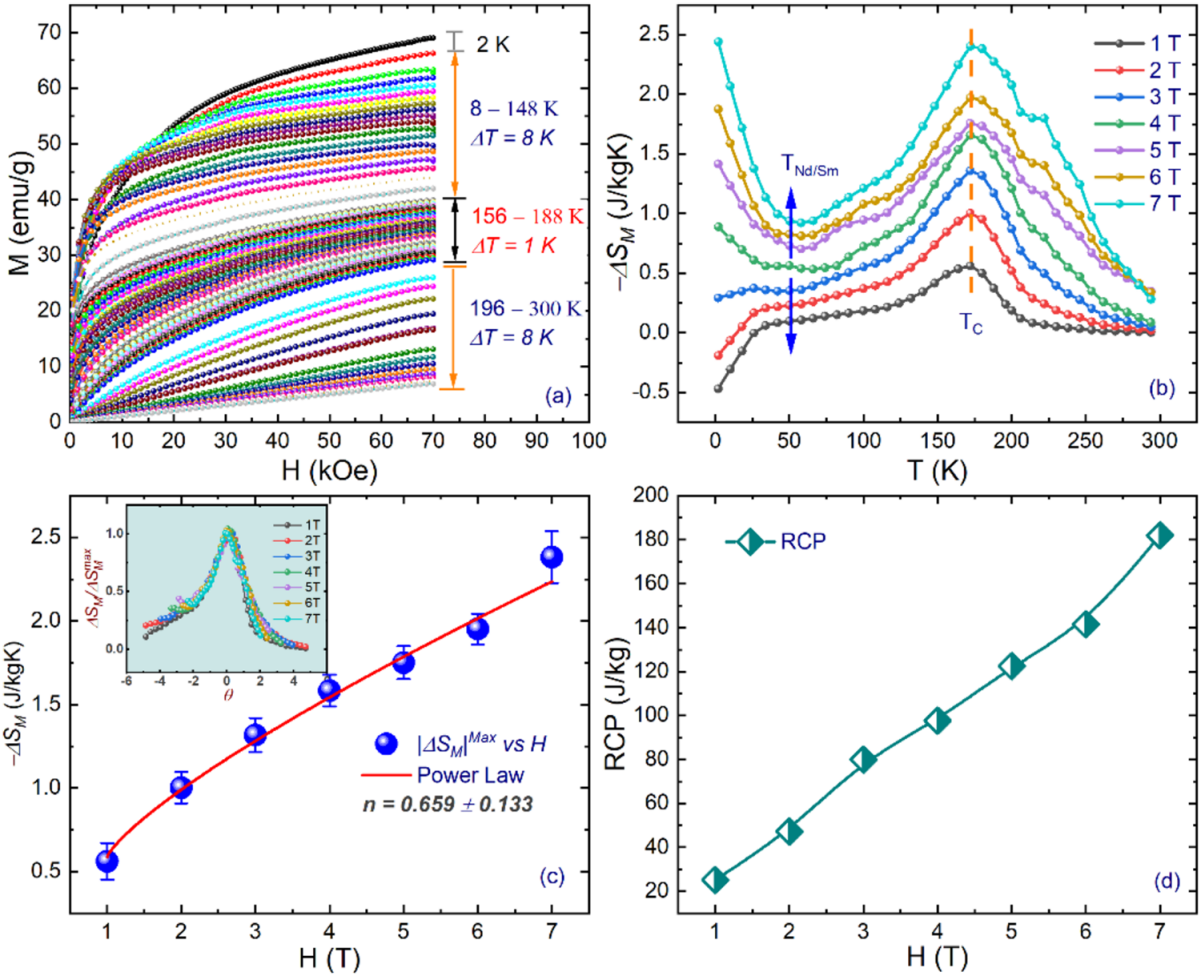
(a) Isothermal magnetization curves at different temperatures from 2–156 K ≤ Δ*T* = 8 K ≤ 196–300 and 156 K ≤ Δ*T* = 1 K ≤ 188 K (critical point analysis). (b) Thermal profile of field-induced magnetic entropy change, −Δ*S*_M_, estimated from isothermal magnetization curves from 2 to 292 K, under various magnetic applied fields. (c) H dependence of maximum magnetic entropy change (−Δ*S*_M_^max^). The inset represents the plot of normalized (Δ*S*_M_/Δ*S*_M_^max^) as a function of reduced temperature θ. (d) Relative cooling power RCP as a field (H).

The magnetocaloric effect consists of the magnetization response to the temperature variation under an applied magnetic field. Usually, the MCE is quantified by magnetic entropy change (Δ*S*_M_) estimated through the analysis of isotherms (*M*–*H*) at several temperatures. Figure 2a illustrates the *M*–*H* curves collected with applied fields up to 7 T and various temperatures between 2 and 300 K. The curves show a typical saturation trend at low temperatures with a transition to the PM state (*T* > *T*_C_) as the temperature increases up to 300 K. The Δ*S*_M_ under a change in magnetic field can be evaluated from the Maxwell relation as follows:
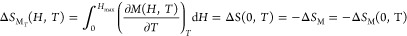
2

Notwithstanding, due to the discrete interval of *T* and *H* under magnetic measurements, the above equation has been approximated as
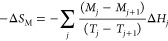
3where *M*_*j*_ and *M*_*j*+1_ are the magnetization values measured in the applied field *H*_*j*_ at temperatures *T*_*j*_ and *T*_*j*+1_, respectively.^40−41^

Figure 2b shows the temperature dependence of −Δ*S*_M_ for different magnetic fields. It can be observed that there is a broad peak centered at *T*_C_ ≈ 180 K characteristic of the conventional MCE, which confirms the FM-PM order transition in our system. This pronounced peak at 180 K presents a maximum entropy change −Δ*S*_M_^max^ = 2.38 J kg^–1^ K^–1^ at 7 T, which is lower than other Ni/Mn-based DPs^42−43,44^ findings and somewhat larger than those of SmCaCoMnO_6_^41^ and La_2–*x*_Sr_*x*_CoMnO_6_^45^ (discussed later). Interestingly, at a low temperature (*T* < 50 K), a negative −Δ*S*_M_ behavior is observed for the applied field up to 2 T. As the applied field is increased, it transits to a positive MCE state in the interval of 3–7 T (blue arrows for eye guide). This abrupt rise in −Δ*S*_*M*_ at low temperature (for a field greater than 4 T) has been attributed to the rare-earth ions ordering.^40,46^ The observed phenomenon is attributed to the AFM ordering of the (Nd^3+^, Sm^3+^) moments. Prior studies indicate that rare-earth sublattices, particularly those containing Nd and Sm, can undergo AFM coupling at low temperatures, significantly influencing magnetization behavior in weak fields.^25^ In our system, complex magnetic interactions emerge between the rare-earth (Nd/Sm) and transition metal (Ni–Mn) sublattices. At low fields, Nd/Sm moments align antiferromagnetically with the Ni/Mn sublattice, contributing to a negative Δ*S*_M_. However, as the field strength increases, these moments gradually reorient ferromagnetically, leading to a positive Δ*S*_M_, characteristic of a metamagnetic-like transition.^35,47^ This AFM interaction competes with the intrinsic FM component, reducing the overall magnetization at low temperatures under weak fields. The disappearance of this effect at higher temperatures supports the role of rare-earth ordering as thermal fluctuations suppress AFM coupling. A similar interplay of AFM and FM interactions, resulting in field-dependent entropy variations, has been observed in other perovskite systems such as Nd_2_NiMnO_6_^44^ and Y_2_NiMnO_6_.^48^ Furthermore, a small peak with an asymmetrical shape at *T* ≈ 214 K is visible for fields greater than 4 T, which could be associated with disorder- or magnetic frustration-like behaviors or emerging Griffiths phase.^40,49^

Notably, the observed *–*Δ*S*_M_ value remains below the theoretical limit (−Δ*S*_M–limit_), which is generally estimated from the contribution of uncoupled *RE* ions. The theoretical limit is expressed as −Δ*S*_M–limit_ = *R*·ln(2*J*+1), where *R* represents the universal gas constant and *J* accounts for the half-filled 4f orbital of Nd^3+^/Sm^3+^ ions, characterized by a high-spin state of *J* = 9/2 and 5/2,^14,50^ respectively. The calculated −Δ*S*_M–limit_ is 22.52 J kg^–1^ and K^–1^ for NdSmNiMnO_6_. This observed disparity is likely associated with additional internal entropy loss originating from phonon contributions, as well as constraints on the Δ*H* values in the measurements.^51,52^ Plausibly, the division of the *A*-site by Nd and Sm might have caused a modification of the global bandwidth, hence deviating the Ni–O–Ni/Mn bond angle <180° and the Ni/Mn–O lengths, as observed in XRD analysis, contrary to Nd_2_NiMnO_6_.^39^ Consequently, the electron-spin coupling in the system will increase, enhancing the FM in the NdSmNiMnO_6_.^53^ Generically, the exponent *n* characterizes the field dependence of the magnetic entropy change (Δ*S*_M_) in magnetocaloric materials, following the relation Δ*S*_M_ ∝ H^*n*^.^28^ Its value provides insight into the nature of the magnetic phase transition. According to the study by Law et al. (2018),^54^ a maximum *n* value exceeding 2 is indicative of a first-order phase transition (FOPT), serving as a quantitative fingerprint for such transitions. In contrast, for second-order phase transitions (SOPTs), *n* typically assumes lower values. In our analysis, we determined *n* = 0.66(13) (see Figure 2c for the curve), aligning closely with the mean-field prediction of *n* = 2/3.^52,53^ This suggests that our material undergoes a SOPT, equivalent to reports of other perovskite systems. For instance, in La_0.6_Ca_0.3_Sr_0.1_MnO_3_, the exponent *n* was found to be approximately 0.58,^55^ consistent with a SOPT behavior. Additionally, studies on Ag-doped manganites revealed *n* values around 0.669,^56^ further corroborating the second-order nature of the phase transitions in these materials. These consistent findings across different studies underpin the reliability of using exponent *n* as a criterion for determining the order of magnetic phase transitions. The proximity of our *n* value to 2/3 supports the classification of the observed transition as second-order, providing valuable insights into the magnetic properties of our material.

To further clarify the characteristics of the FM phase transition, we examine the MCE by employing a normalizing universal scaling law.^28^ All curves were constructed phenomenologically by scaling all −Δ*S*_M_ curves against their respective maximum values (−Δ*S*_M_^max^), expressed as (Δ*S*_M_/Δ*S*_M_^max^). For this, the reduced temperature *θ±* is defined as follows:^28^
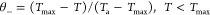
4
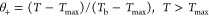
5where *T*_max_ denotes the temperature at −Δ*S*_M_^max^, whereas *T*_a_ and *T*_b_ are the temperatures of two reference points (below and above *T*_C_) determined by . As highlighted in the inset of Figure 2c, all of the rescaled Δ*S*_M_/Δ*S*_M_^max^ (θ) curves collapse into a unified curve. The observed single collapsed pattern at *θ > 0* closely confirms the characteristic behavior of materials undergoing typical FM second-order phase transitions (FM–SOPT). However, the noncollapsing character at *θ < 0* is attributed to the uncertainty in the normalizing or a possible first-order phase transition (FOPT) due to the presence of ASD.

Normally, the relative cooling power (RCP) parameter is used to evaluate the cooling efficiency of the materials. RCP can be determined from −Δ*S*_M_(*T*) curves^14,57^ as
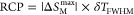
6where δ*T_FWHM_* = *T*_Hot_ – *T*_Cold_ is the full width at half-maximum of the change in magnetic entropy. The RCP value as a function of the magnetic field *H* is displayed in Figure 2d for NdSmNiMnO_6_. Significantly, RCP demonstrated an increasing tendency with increasing Δ*H*, which obeys the power law RCP ≈ H^*m*^ (*m* is an exponential scaling factor). The estimated value of RCP in the NdSmNiMnO_6_ sample increases from ∼25 to ∼182 kg^–1^ K^–1^ when the applied field changes from 1 to 7 T. The remarkable discovery of MCE properties in pure Gd by Brown has set the benchmark for ideal MCE materials,^58^ and according to Phan and Yu, optimal MCE materials should exhibit large magnetic entropy change (−Δ*S*_m_) and adiabatic temperature change (Δ*T*_a_*d*), a Curie temperature near room temperature (∼300 K), a broad operating temperature range (e.g., 10–80 K), near-zero magnetic and thermal hysteresis, small lattice entropy, high thermal conductivity, and high chemical stability.^59^ Recent studies highlight that perovskite manganites offer advantages such as tunable Curie temperature, chemical stability, and low hysteresis, making them attractive for magnetic cooling applications.^60^Table 2 presents a comparative analysis of NdSmNiMnO_6_ with well-known magnetocaloric materials, including those in which its performance exceeds other reported values. Compared to high-performance perovskite materials, the reported maximum magnetic entropy change in NdSmNiMnO_6_ (−Δ*S*_M_^max^ = 2.38 J kg^–1^ K^–1^ at 7 T) is significantly lower than in Gd-,^61^ Gd_5_SiGe_2_-,^59^ Ln_0.5_Ca_0.5_MnO_3_- (Ln = Dy,Gd),^62^ and MnFeP-based alloys.^47^ Notably, Tb_2_FeCrO_6_ achieves −Δ*S*_M_ = 12.9 J kg^–1^ K^–1^ under the same field conditions,^63^ while SmFeO_3_ perovskites with Mn substitution exhibit a broader operational range with higher entropy changes.^64^ Similarly, La(Fe_0.98_Co_0.02_)_11.7_Al_1.3_^65^ demonstrates −Δ*S*_M_ ∼ 10.6 J kg^–1^ K^–1^ at 0–5 T, surpassing NdSmNiMnO_6_ in absolute entropy change. Despite this, the relative cooling power (RCP) remains a crucial parameter for evaluating refrigeration efficiency. NdSmNiMnO_6_ exhibits a notable increase in RCP from ∼25 to ∼182 J kg^–1^ K^–1^ as the applied field varies from 0–1 T to 0–7 T, indicating a compensatory mechanism, likely due to an extended temperature span of the MCE. A comparison of the relative cooling power and magnetic entropy change values for the investigated NdSmNiMnO_6_ and other reported materials is given in Table 2. This RCP surpasses materials like La_0.5_Nd_0.2_Ca_0.3_MnO_3_ and La_0.7_Ca_0.3_MnO_3_,^66^ reinforcing the viability of NdSmNiMnO_6_ for practical applications. While NdSmNiMnO_6_ underperforms in absolute entropy change, its superior RCP and promising thermal response highlight its potential. Further optimization in composition and microstructure could enhance its overall magnetocaloric performance, making it a viable candidate for future magnetic refrigeration technologies.^14,48,53,57^

**Table 2:** Comparative Analysis of **NdSmNiMnO**_**6**_ with Well-Known Magnetocaloric Materials

material	–Δ***S***_**M**_^**max**^ (J kg^–1^ K^–1^)	RCP (J kg^–1^)	reference
Gd	10.2	∼410	Tegus^61^
La_0.87_Sr_0.13_MnO_3_	5.80	∼232	Phan & Yu^59^
Gd_5_SiGe_2_	18.4	∼535	Pecharsky et al.^67^
Pr_0.63_Sr_0.37_MnO_3_	2.57–8.52	∼511	Phan et al.^68^
MnFeP_0.45_As_0.55_	18.6	∼500	Tegus et al.^61^
Fe_2_P-based alloy	7.8	∼390	Fujita et al.^47^
La_0.7_Ca_0.3_MnO_3_	1.38	∼41	Wang et al.^66^
Ln_0_._5_Ca_0_._5_MnO_3_(Ln = Dy,Gd)	8.5–22.8	-	Das et al.^69^
((R_0.2_Gd_0.2_Eu_0.2_Dy_0.2_Tb_0.2_)CrO_3_, R = Pr, Nd and Sm)	12.8–13.6	∼143.71–159.03	Zheng et al.^62^
La_0.5_Nd_0.2_Ca_0.3_MnO_3_	2.31	∼60	Wang et al.^66^
La_0.6_Ca_0.4_MnO_3_	5.00	∼135	Phan & Yu^59^
NdSmNiMnO_6_	2.38	∼182	this study

### 3.3 Critical Exponent Analysis

To elucidate the nature of magnetic interactions near the magnetic phase transition temperature *T*_C_ = 177 K, we conducted a comprehensive analysis of the critical exponents for NdSmNiMnO_6_. Primarily, according to the scaling hypothesis, a second-order phase transition (SOFT) is characterized by interconnected critical exponents.^70^ Thus, in the vicinity of SOFT, the correlation length exhibits a divergence given by *ξ = ξ*_0_|(*T* – *T*_c_)/*T*_c_*|*^^*–*^*ν*^, where universal scaling laws govern the spontaneous magnetization, *M*_S_, and the inverse initial magnetic susceptibility, χ_0_^–1^. The critical exponents β (spontaneous magnetization in the absence of a magnetic field), γ (related to initial magnetic susceptibility), and δ (linked to the critical magnetization isotherm) characterize *M*_S_ below *T*_C_, χ_0_^*–*1^ above *T*_C_, and *M*(*H*) at *T*_C_,^71^ as follows:
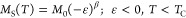
7
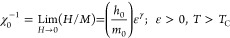
8
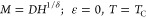
9where ε = (*T* – *T*_C_)/*T*_C_ is the reduced temperature and *h*_0_/*m*_0_, *D*, and *M*_0_ are the critical exponent amplitude of spontaneous magnetization.^72,73^ In this case, the relationship between the variables of *M*(*H*, *ε*), *H*, and *T* that governs the magnetic equation of states is given as
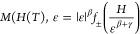
10where the regular functions are *f*_+_ (*T* > *T*_C_) and *f*_–_ (*T* < *T*_C_). We can then define a renormalized term as *m* = *f*_±_(*h*), where *m* ≡ |*ε*|^–β^*M*(*H*, *ε*) and *h* ≡ |*ε*|^–(β+γ)^*H* or |*ε*|−^*βδ*^*H* are the renormalized magnetization and field, respectively. This relation is satisfied when a plot of |*ε*|^–β^*M*(*H*, *ε*) vs |*ε*|^–(β+γ)^*H* with a correct choice of β, γ, and δ collapses onto two universal curves, where one represents *T* < *T*_C_ and the other *T* > *T*_C_ temperature regions, correspondingly.^73−75^

**Figure 3:**
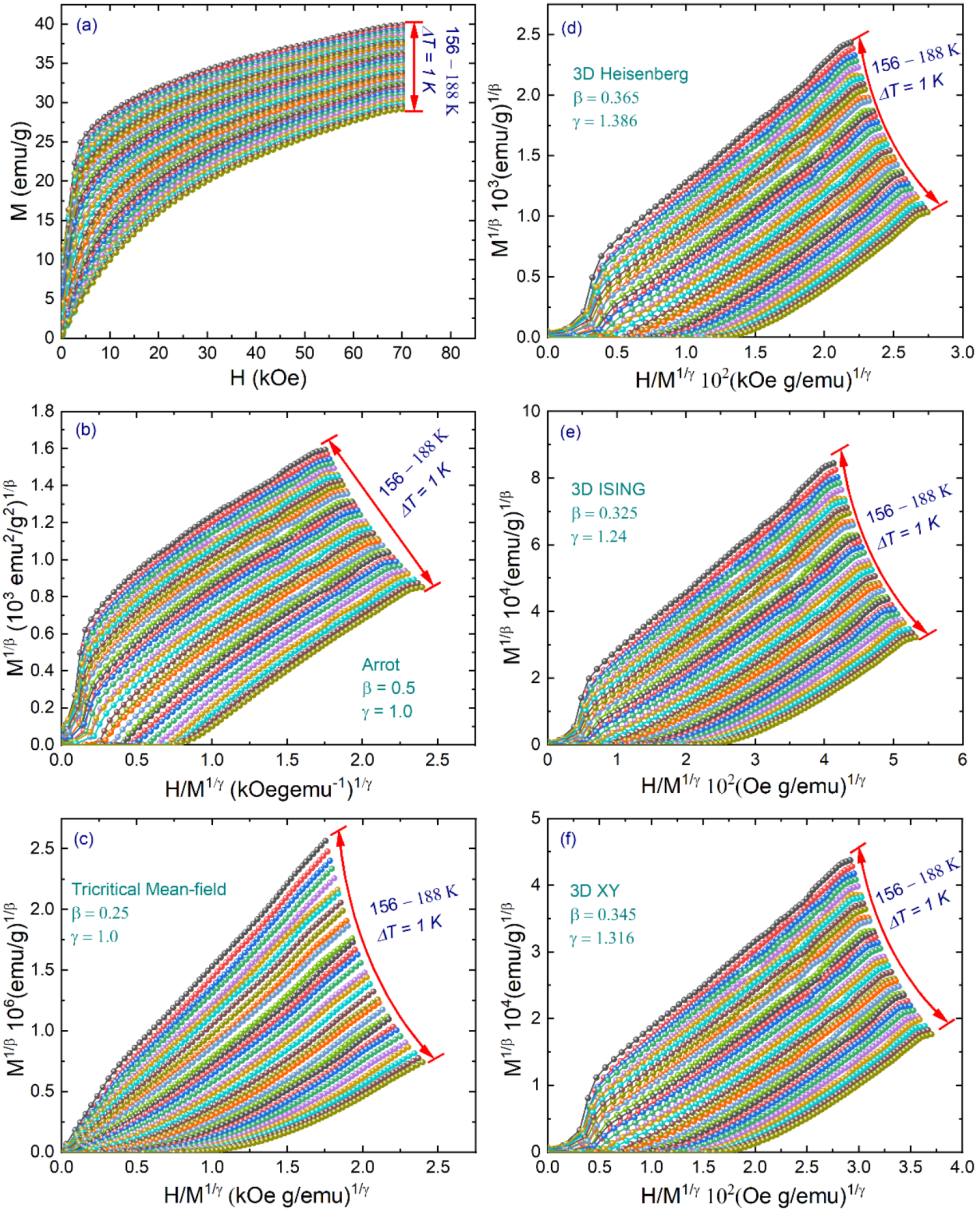
(a) Isothermal magnetization curves at different temperatures between 156 and 188 K with Δ*T* = 1 K. The isotherms plotted as *M*^1/β^ vs (*H*/*M*)^1/γ^ with model parameters from (b) Arrott–Noakes, (c) tricritical mean-field, (d) three-dimensional (3D) Heisenberg, (e) 3D Ising, and (f) 3D XY, respectively.

Figure 3a shows the isotherms collected in the vicinity of *T*_C_ between 156 and 188 K with Δ*T* = 1 K. As usually, we displayed the Arrott–Noakes plot (*M*^*2*^ vs *H*/*M*), which involves mean-field critical exponents *β = 0.5* and *γ = 1.0*(76) in Figure 3b. For a suitable model, around *T*_C_, the isotherm at *T = T*_C_ should pass through the origin, yielding χ_0_^–1^ (*T*) and *M*_S_ (*T*) as intercepts on the *H*/*M* axis and positive *M*^*2*^ axis, respectively. Unlike the hypothesis of the Landau mean-field model, all curves in the Arrott–Noakes plot display a nonlinear trend with a downward curvature, indicating the inadequacy of the mean-field model for NdSmNiMnO_6_. Nevertheless, it is feasible to estimate the order of the magnetic transition using Banerjee’s criterion,^77^ where the slope of the straight line plays a crucial role. A first- or second-order phase transition corresponds to a negative or positive slope, respectively. Thus, the positive slope confirms the SOPT in NdSmNiMnO_6_.

Mostly, the modified Arrott–Noakes plot (MAP) was utilized to probe critical behavior based on other models,^76^ described as
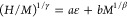
11where *ε* has been defined earlier, *a* and *b* are constants. Figure 3c–f depicts the MAP for the models: tricritical mean-field (β = 0.25, γ = 1.0), 3D Heisenberg (β = 0.365, γ = 1.386), 3D Ising (β = 0.325, γ = 1.24), and 3D XY (β = 0.345, γ = 1.316), respectively. All replotted MAP displays a slight curvature in the low-field region, indicative of the average effect across domains magnetized in diverse directions. Conversely, within the high-field region, all constructions manifest quasilinear profiles, implying potential 3D magnetic behavior within the material.

**Figure 4:**
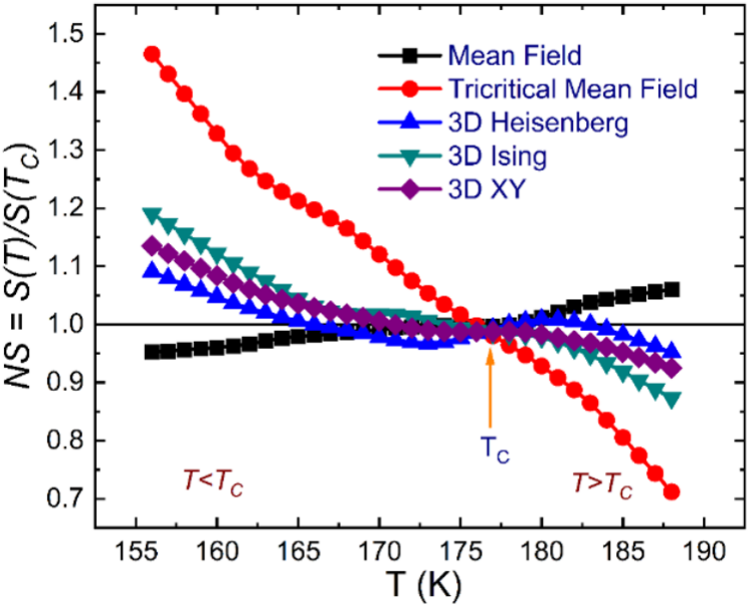
Temperature dependence of the normalized slopes, NS = *S*(*T*)/*S*(*T*_C_*)* for the mean-field, tricritical mean-field, 3D Heisenberg, 3D Ising, and 3D XY models, respectively.

To identify the optimal model, we introduce normalized slopes (NS) defined as NS = *S*(*T*)/*S*(*T*_*C*_*)*, where *S*(*T*) represents the slopes of the MAP curves. The NS(*T*) curves are presented in Figure 4. In an ideal scenario, the MAP plot is expected to yield a set of parallel straight lines, resulting in all NS values being equal to 1.0. However, our observations indicate that the critical behavior of NdSmNiMnO_6_ deviates slightly from adherence to these universality classes. We noted that the NS approaches the mean-field model below *T* < *T*_C_, aligned with the nearly isotropic magnetic nature evident at low temperatures. In contrast, the NS for the 3D Heisenberg model exhibits optimal agreement above *T* > *T*_C_, signifying that the critical behavior of the NdSmNiMnO_6_ system may not conform to a singular universality class, which is in contrast to other DP compounds.^37,48,78^

**Figure 5:**
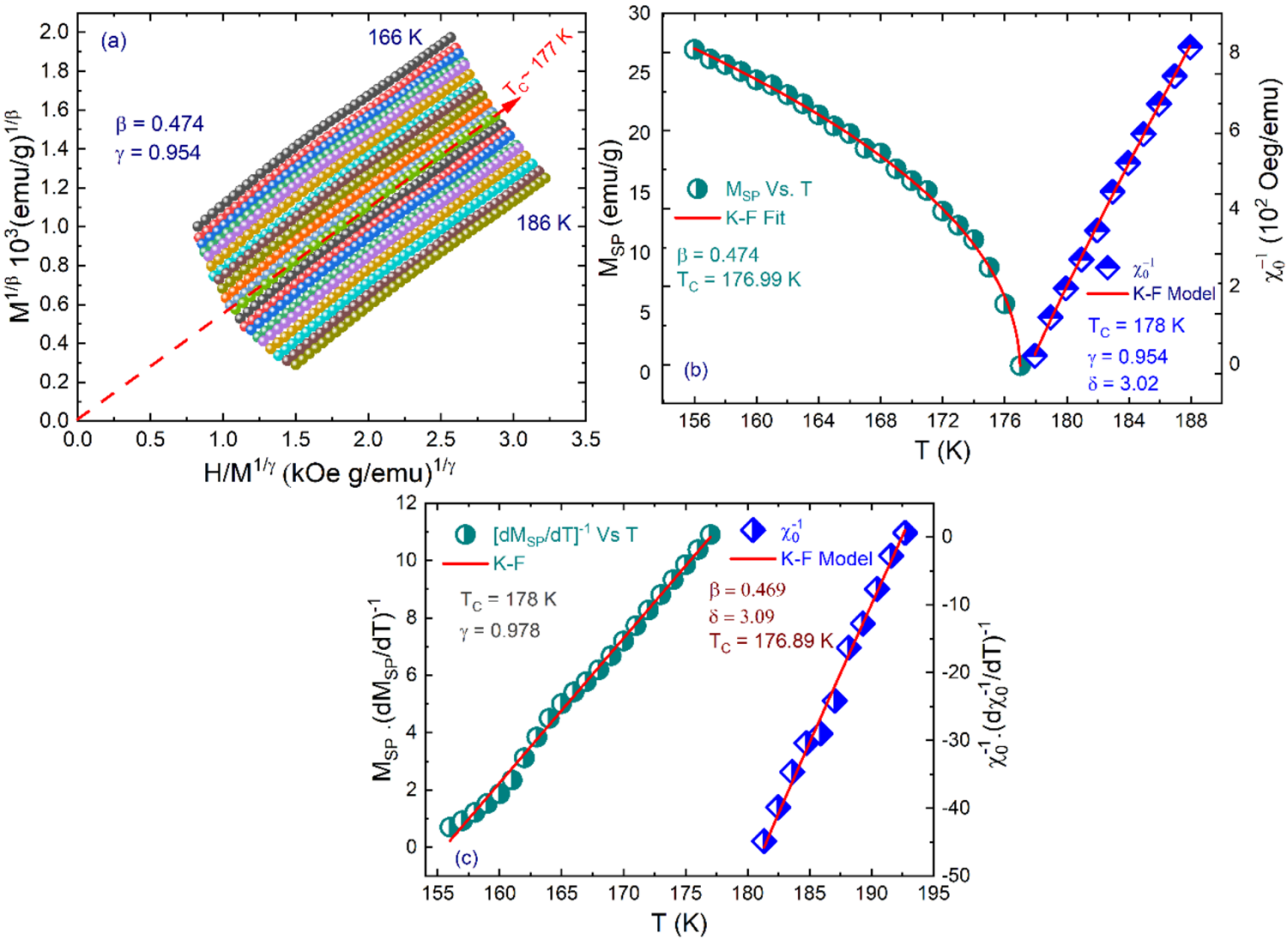
(a) Modified Arrott plot of isotherms with β = 0.474 and γ = 0.954. (b) Temperature dependence of spontaneous magnetization *M*_s_ (left) and the inverse initial magnetic susceptibility χ_0_^–1^(*T*) (right), which are obtained from the high-field extrapolation of the modified Arrott plot. The *T*_C_ and exponent values are deduced by fitting eqs 8 and 9 (red curves). (c) Kouvel–Fisher plot for the temperature dependence of the spontaneous magnetization *M*_s_ (left) and the inverse initial magnetic susceptibility χ_0_^–1^(*T*) (right). The *T*_C_ and critical exponents are obtained from the linear fits (red lines).

**Figure 6:**
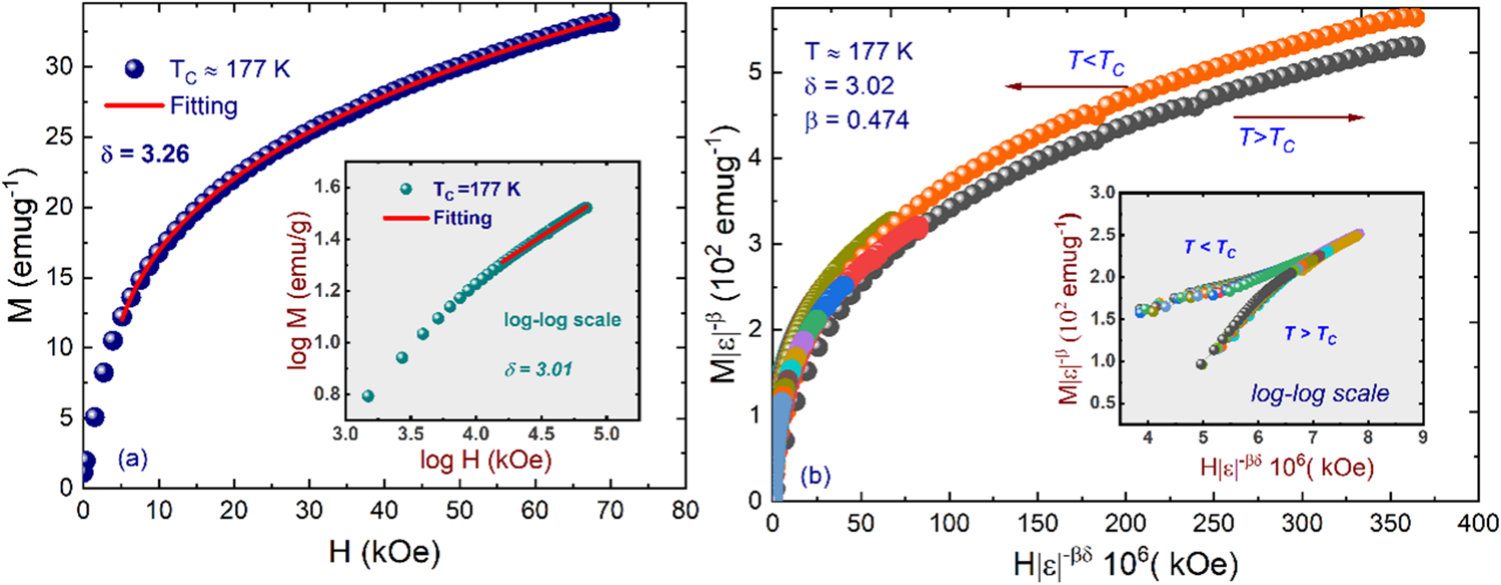
(a) *M*(*H*) curve at *T*_C_*∼* 177 K. Inset: the same plot in log–log scale with a solid fitting curve in the high-field region. (b) Scaling plots of renormalized magnetization *m* = *M*|*ε*|^–β^ vs renormalized field *h* = *H*|*ε*|^–βδ^ below and above *T*_C_ for NdSmNiMnO_6_. The inset represents the same plots in the log–log scale.

Hence, we employ an iterative approach to precisely determine the critical exponents β and γ.^40,74^ By linear extrapolation from the high-field region to the intercepts with the axes *M*^1/β^ and (*H*/*M*)^1/γ^, reliable values of *M*_S_(*T*) and χ_0_^–1^(*T*) are obtained. Subsequently, fitting the data using eqs 8 and 9 yields a set of β and γ. These values are then employed to reconstruct MAP, leading to the generation of new *M*_SP_(*T*) and χ_0_^–1^(*T*). The process is repeated, refining the values of β and γ until stability is achieved. As depicted in Figure 5a, a series of parallel straight lines in the high-field regime is attained through converged β and γ values. Figure 6b displays the final temperature dependence of *M*_SP_(*T*) (left) and χ_0_^–1^(*T*) (right), which are obtained from the high-field MAP curve extrapolation. The fitting yielding critical exponents β = 0.474 with *T*_C_ = 177 K, and γ = 0.954 with *T*_C_ = 178 K, are attained.

Alternatively, for a more precise determination of the critical exponents and *T*_C_, we have examined the *M*_SP_(*T*) and χ_0_^–1^(*T*) data using the Kouvel–Fisher (K–F) plot analysis:^72^
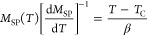
12
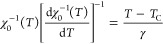
13where  and  are linear in the temperature with the slopes 1/β and 1/γ, and the intercepts signify −*T*_C_/*β* and *–T*_C_/*γ*, accordingly. The critical exponents and *T*_C_ determined through the K–F method are found to be β = 0.469 with *T*_C_ = 176.89 K and γ = 0.978 with *T*_C_ = 178 K (see Figure 5c).

Remarkably, these critical exponent values and *T*_C_ closely agree with those computed by using the mean-field model and 3D Heisenberg. This congruence suggests that the derived values of the critical exponents are self-consistent and unequivocal. The critical exponent δ, related to the magnetization isotherm at *T*_C_ through eq 10 with *D* representing the critical amplitudes, can be ascertained by fitting the high-field slope of log_10_(*M*) vs. log_10_(*H*) plot at *T*_C_. Figure 6a illustrates the M(*H*) curve at *T*_C_ ∼ 177 K, with the inset depicting the same plot in the log–log scale. Fitting the *M*(*H*) curve at *H* > 5 kOe yields δ = 3.26 and δ = 3.01 for the log–log fits, respectively. Additionally, the critical exponents obtained through scaling analysis adhere to the Wisdom scaling relation,^79,80^ as δ = 1 + γ/β. Utilizing the determined values of β and γ from the MAP and the K–F method yielded δ values of 3.02 and 3.09, respectively, proximate to the aforementioned δ value. This underscores the reliability of the critical exponents derived from the magnetization data and their agreement with the scaling hypothesis.

Based on the scaling hypothesis, the credibility of the acquired critical exponents was affirmed through the magnetic equation of state in the asymptotic critical region, as clarified earlier in eq 11. To affirm this, we constructed a plot of |*ε*|^–β^*M*(*H*, *ε*) vs |*ε*|^–(*βδ*)^*H* with the β, δ, and *T*_C_ values derived through the K–F technique. Figure 6b displays the *M*|ϵ|^–β^ vs *H*|ϵ|^–βδ^ curves in the scaled data at *T* < *T*_C_ and *T* > *T*_C_, and the inset presents the same data in log–log coordinates for clarity. All magnetization curves collapse into two distinct branches (*T* < *T*_C_ and *T* > *T*_C_), implying the accuracy and consistency of the critical exponents and *T*_C_ values for NdSmNiMnO_6_ following the scaling hypothesis. The typically subtle departure from the scaling curves noted in the low-magnetic-field region is attributed to the reconfiguration of the magnetic domains. In this regime, magnetic moments exhibit an incomplete alignment with the applied magnetic field.

Finally, as is well-established, the universality class governing the magnetic transition in homogeneous magnets is dependent upon the exchange interaction *J*(*r*). Analysis using renormalization group theory suggests that the decay of interaction with distance *r* can be described as
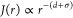
14where *d* represents spatial dimensionality and σ denotes the range of interaction, assuming a positive value. In the context of 3D isotropic materials (*d = 3*), this relationship translates to *J*(*r*) ∝ *r*^–(3+σ)^. If σ > 2, it indicates a more rapid decrease than *r*^*–5*^; hence, the applicability of the 3D Heisenberg model (β = 0.365, γ = 1.336, and *d* = 4.8) emerges. For σ = 3/2, corresponding to the mean-field model (β = 0.5, γ = 1.0, and *d* = 3), this signifies that *J*(*r*) decreases more gradually than *r*^*–4.5*^. Within the intermediate range, i.e., *J*(*r*) ∝ *r*^–(3+σ)^ with 3/2 ≤ σ ≤ 2, the exponent aligns with a distinct universality class reliant on the specific value of σ. In this range, both the tricritical mean-field theory and the 3D Ising model are plausible. Fischer et al.^81^ propose a relationship between the exponent γ and the interaction range σ as
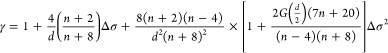
15where ,  and *n* is the magnetic spin dimensionality given as *n* = 1 + (β – 1/β + γ).^40^ From β and γ values of the scaling analysis, we deduced self-consistent critical exponents *n* = 0.632, which agrees with the previously mentioned mean-field experimental value, as depicted in Figure 5c. Therefore, using eq 15 and the obtained value of γ = 0.954 in this study, we calculated σ = 1.629, indicating that the magnetic interaction distance decays with *J*(*r*)∝ *r*^–4.629^, which is close to other ferromagnetic perovskite systems.^48,73^ Indeed, *J*(*r*) lies between the characteristics of the 3D Heisenberg model and the mean-field model, suggesting that the critical behavior of NdSmNiMnO_6_ can be attributed to the competition between long- and short-range magnetic interactions rather than purely long-range interaction regimes.^81^ Nonetheless, its closer resemblance to the mean-field model suggests that the dominant spin interactions are of a long-range magnetic nature. Furthermore, we note that our values of β and γ obtained from the modified Arrott plot align closely with the mean-field predictions but deviate significantly from the Heisenberg model, which suggests that the system does not exhibit a purely short-range 3D Heisenberg-like transition. Similar deviations have been observed by Pramanik et al., such as in Pr_0.5_Ca_0.5_MnO_3_,^82^ where long-range interactions play a significant role in determining the critical behavior. Hence, the exchange distance *J*(*r*) analysis suggested that the long-range magnetic interaction coupling between ordered Ni–Mn ions at BB′ sites dominates near *T_N_*, which is responsible for the magnetic nature of the NdSmNiMnO_6_ double perovskite. Furthermore, under the correlation length critical exponent relation, ϑ = γ/σ (i.e., ϑ = 0.584), the critical exponents, α = (2 – *ϑd*), β = (2 – α – γ)/2, and δ = (1 + γ/β), are determined, yielding values of approximately 0.243, 0.401, and 3.38, respectively. Notably, these calculated values closely align with the experimentally observed values in our study.

The critical exponents obtained for NdSmNiMnO_6_ (β = 0.469, γ = 0.978, δ = 3.09) suggest a behavior intermediate between the 3D Heisenberg model (β ≈ 0.365, γ ≈ 1.386, δ ≈ 4.8) and the mean-field model (β ≈ 0.5, γ ≈ 1.0, δ ≈ 3.0).^83,84^ Such deviations are commonly observed in complex magnetic oxides, where multiple competing interactions influence the critical exponents. A key factor contributing to this behavior is the competition between nearest-neighbor (NN) and next-nearest-neighbor (NNN) interactions, a phenomenon reported in perovskite-based oxides. In NdSmNiMnO_6_, the coexistence of FM and AFM interactions arising from double-exchange (DE) and superexchange (SE) mechanisms alters the effective correlation length.^85^ For instance, La_0.67_Ca_0.33_MnO_3_ and Nd_0.5_Sr_0.5_MnO_3_ exhibit competing interactions that produce deviations from conventional universality classes.^59^ Moreover, the presence of a Griffiths-like phase, characterized by the persistence of local magnetic clusters above the transition temperature due to disorder and inhomogeneity, could induce this behavior.^86^ In the case of NdSmNiMnO_6_, the slightly elevated β value (0.469) suggests an inhomogeneous ordering process, consistent with the study by Bray et al. for perovskite oxides, where structural disorder and phase separation broaden the critical region.^87^ The presence of such magnetic clusters results in modified scaling behavior, contributing to the deviation from classical universality classes. Further, the universality class of a phase transition is deterministic by the effective interaction range, as proposed by the renormalization theory.^88^ Particularly, the crossover interaction is influenced by bond-angle distortions, spin–orbit coupling, and the cooperative Jahn–Teller effect, modifying the interaction range. In our case, the observed bond-angle deviations and octahedral tilting in NdSmNiMnO_6_ suggest that the magnetic SE pathways between Ni and Mn ions are significantly affected, influencing DE and SE interactions.^17,89^ In aristo-type cubic perovskites, magnetic superexchange follows the GKA rule, with 180° Ni–O–Mn bonds favoring strong FM coupling and AFM double exchange occurring for partially delocalized electrons. However, the observed Ni–O–Mn bond angles (∼150.5°) deviate from 180°, weakening direct AFM superexchange while allowing for competing FM–DE interactions, a feature of magnetically frustrated systems.^90^ The estimated octahedral rotation angles (γ, φ, Φ, and Θ) further support this magnetic competition hypothesis, i.e., the significant tilting of NiO_6_ and MnO_6_ octahedra modifies orbital overlap, altering the Ni^2+^–O–Mn^4+^ SE-like interactions, which can lead to the emergence of magnetic phase coexistence.^91^ Several studies have reported that octahedral perovskite distortions result in noncollinear magnetic ordering, magnetic phase separation, and spin-glass-like behavior due to competing interactions.^92−94^ In NdSmNiMnO_6_, the combination of bond length/bond-angle variations, octahedral tilting, and local lattice distortions likely contributes to frustrated magnetic behavior observed below *T* < *T_N_* in the MCE analysis, explaining the observed complex critical exponents and intermediate universality class. Also, our critical exponents, tested using the Wisdom scaling equation [δ = 1 + γ/β = 3.09], the Arrott plots, and Kouvel–Fisher analysis, demonstrate that the exponents are self-consistent. On the other hand, magnetocrystalline anisotropy and spin fluctuations play a critical role in determining the critical behavior of NdSmNiMnO_6_. In systems with strong anisotropic interactions, critical exponents often deviate from classical models due to anisotropic spin fluctuations and spin–orbital coupling effects.^87^ This is evident in our MCE analysis that the Nd/Sm sublattice flipped from negative to positive entropy change in the temperature range below ∼50 K as the field increases from 0–3 to 0–7 T. The interplays between spin, charge, and orbital degrees of freedom likely influence the scaling behavior, where the presence of unquenched orbital moments and anisotropic exchange interactions can lead to modified critical behavior.^47^ Hence, NdSmNiMnO_6_ and the observed exponents suggest a scenario where short-range spin interactions dominate at lower temperatures, but longer-range interactions contribute near the Curie temperature, leading to deviations from standard universality classes.

## 4 Conclusions

In summary, we have successfully synthesized the NdSmNiMnO_6_ double perovskite and systematically studied its crystal structure, magnetocaloric effect, and critical behavior. The monoclinic structure of NdSmNiMnO_6_ identified from the XRD pattern and refined by Rietveld demonstrated that Nd/Sm shared the same crystallographic A-site, whereas Ni/Mn cations are distinctly distributed at the octahedron BB′ sites. The −Δ*S*_M_^max^ and RCP at the applied field of 0–7 T are found to be 2.38 and 182 J/kg K, respectively, suggesting that NdSmNiMnO_6_ can be considered a promising magnetic refrigerant. The critical exponents β, γ, and δ obtained by different methods indicate a proximity to the conventional universality class of the mean-field model in the limit of *T* < *T*_C_ and a 3D Heisenberg-like phenomenon at *T* > *T*_C_. Finally, the exchange distance *J*(*r*) analysis suggested that in NdSmNiMnO_6_, the observed exponents suggest a scenario where short-range spin interactions dominate at lower temperatures, but longer-range interactions contribute near the Curie temperature, leading to deviations from standard universality classes.
